# Nosokomiale Pneumonien und beatmungsassoziierte Krankenhauserreger: Teil 2: Therapie

**DOI:** 10.1007/s44179-022-00108-9

**Published:** 2022-11-09

**Authors:** Holger Flick, Maria Hermann, Matthias Urban, Michael Meilinger

**Affiliations:** 1 ÖGP-Arbeitskreis „Pulmonale Infektionen und Tuberkulose“, Wien, Österreich; 2 Klinische Abteilung für Pulmonologie, Universitätsklinik für Innere Medizin, LKH-Univ. Klinikum Graz, Medizinische Universität Graz, Graz, Österreich; 3 Abteilung für Innere Medizin und Pneumologie, Klinik Floridsdorf, Wien, Österreich; 4 ÖGP-Arbeitskreis „Beatmung und Intensivmedizin“, Wien, Österreich; 5 Karl Landsteiner Institut für Lungenforschung und pneumologische Onkologie, Wien, Österreich

## Einleitung

Nosokomiale Pneumonien gehen für die betroffenen Patient*innen mit einem erhöhten Sterblichkeitsrisiko einher und sind mit hohen Kosten für das Gesundheitswesen assoziiert. Die letzten internationalen Leitlinien wurden 2016/2017 veröffentlicht [1, 2]. Dieser zweiteilige Übersichtsartikel beschreibt die wesentlichen diagnostischen und therapeutischen Standards und evidenzbasierte Trends im klinischen Management. Berücksichtigt werden neue Erkenntnisse im Bereich Epidemiologie, Erregerspektrum und Diagnostik (Teil 1) sowie Therapie und neue antimikrobielle Substanzen zur Behandlung multiresistenter Erreger (Teil 2).

Teil 1 dieses Übersichtsartikels wurde in den *Anästhesie Nachrichten*, Ausgabe 3/2022 publiziert [3] und steht als DFP-Fortbildung online unter www.pains.at [4] oder auf www.meindfp.at [5] zur Verfügung.

## HAP, VAP, Sepsis und septischer Schock

Der Terminus HAP („hospital-acquired pneumonia“ – im Krankenhaus erworbene Pneumonie) umfasst alle Pneumonie-Formen, welche frühestens 48 h nach stationärer Aufnahme auftreten und deren Inkubationszeit nicht in den Zeitraum vor der Krankenhausaufnahme zurückreicht. Gram-negative Enterobakterien und *Staphylococcus aureus* spielen eine wesentliche Rolle als ursächliche Erreger.

### Wichtig.

Bestehen zusätzlich Risikofaktoren für das Vorhandensein multiresistenter Erreger (MRE), sind diese ebenfalls zu bedenken. Liegt eine relevante Immunsuppression vor, ist auch mit opportunistischen Virus- und Pilzinfektionen zu rechnen.

Bezüglich den Details zum Erregerspektrum der HAP und zur rationalen HAP-Diagnostik verweisen wir auf Teil 1 dieses Übersichtsartikels [3]. Pneumonien nach einem stationären Krankenhausaufenthalt in den letzten 90 Tagen können ebenfalls als HAP interpretiert werden oder als „CAP mit erhöhtem Risiko für nosokomiale beziehungsweise (bzw.) multiresistente Erreger“ entsprechend der S3-Leitlinie zur ambulant erworbenen Pneumonie (CAP: „community-acquired pneumonia“) von 2021 [6, 7].

### Wichtig.

Bei schwerer HAP kann zusätzlich eine Sepsis und im Extremfall ein septischer Schock vorliegen. Septische HAP-Patient*innen wie auch die HAP auf Intensivstation („intensive care unit“, ICU) und speziell die beatmungsassoziierte Pneumonie („ventilator-associated pneumonia“, VAP) haben eine besonders schlechte Prognose. Jegliche Therapieverzögerung oder eine initial fehlgezielte Therapie ist mit einer Erhöhung des Letalitätsrisikos assoziiert [8, 9].

## Empirische HAP-Therapie

Obwohl der ursächliche HAP-Erreger und dessen Resistenzprofil initial oft unbekannt sind, muss wie auch bei anderen schweren Infektionen zeitnah eine der Situation angepasste evidenzbasierte und mit hoher Wahrscheinlichkeit effektive antimikrobielle Therapie eingeleitet werden. Diese wird als kalkulierte oder empirische Therapie bezeichnet.

### Wichtig.

Eine adäquate empirische HAP-Therapie verbessert signifikant den klinischen Behandlungserfolg, ist nebenwirkungsärmer, kostengünstiger und verhindert unnötige Resistenzentwicklungen [1, 10, 11, 12, 13]. Im Gegensatz dazu kann eine ungezielte und im Endeffekt oft nicht indizierte empirische „Breitband“-Therapie (unkritisches „overtreatment“, beispielsweise bezüglich Methicillin-resistenter* Staphylococcus aureus* – MRSA – oder Erreger mit „extended spectrum“ Betalaktamasen – ESBL) sogar mit einer erhöhten Mortalität assoziiert sein und das Risiko für Resistenzentwicklungen unnötigerweise erhöhen [14].

## Allgemeine Grundregeln der empirischen HAP-Therapie

Im HAP-Management sind essenzielle Grundprinzipien zu beachten, welche die Basis für eine möglichst erfolgreiche Behandlung darstellen. Diese werden im Folgenden kurz umrissen (wir verweisen ergänzend auch auf Teil 1):

- Vor Beginn oder Umstellung der antimikrobiellen Therapie adäquates Material für die mikrobiologische Diagnostik gewinnen (respiratorisches Sekret, Blutkulturen, EDTA-/Citrat-Blut oder Serum für beispielsweise spezifische Virus‑/Pilzdiagnostik, Urin für *Legionella*-/Pneumokokken-Schnelltest).
- Die empirische Therapie erfasst das zu erwartende Spektrum, spart aber unwahrscheinliche Pathogene/Resistenzen aus (weder „undertreatment“ noch „overtreatment“). Die antimikrobielle „Bandbreite“ ist dabei abhängig von Schweregrad und Akuität der Infektion, dem spezifischen Risikoprofil der Patient*innen und der aktuellen lokalen epidemiologischen Situation.
- Eine empirische Kombinationstherapie ist nur bei hohem Risiko für MRE oder *Pseudomonas* notwendig. Dabei ist der Begriff „hohes Risiko“ nicht klar definiert, das Risiko steigt aber mit der Zahl der erfüllten Risikofaktoren und der Schwere der vorliegenden Infektion (Tab. 1).
- Bei der Auswahl antimikrobieller Substanzen ist auf potenzielle Nebenwirkungen und individuelle Risikofaktoren Rücksicht zu nehmen (Beispiel: Vorsicht mit Aminoglykosiden bei älteren Patient*innen oder bereits reduzierter glomerulärer Filtrationsrate – GFR). Zu erwartende Nebenwirkungen werden durch regelmäßiges aktives Screening frühzeitig erfasst (siehe unten).
- Substanzen mit einer lokalen Resistenzrate für relevante Erreger von > 10 % sind für eine Monotherapie nicht geeignet.
- Bei antimikrobieller Vorbehandlung sollten möglichst Substanzen aus einer anderen Antibiotika-Klasse verwendet werden (Beispiel: nach Piperacillin/Tazobactam folgt eine Therapie mit beispielsweise Carbapenem oder Cephalosporin).
- Antiinfektiva müssen ausreichend hoch dosiert, initial intravenös appliziert und die Dosis je nach Substanz gegebenenfalls der Nierenfunktion angepasst werden.
- Die Wirksamkeit der empirischen Therapie wird nach circa 72 h reevaluiert. Bei ausbleibender klinischer Besserung sind Komplikationen (beispielsweise Empyem, Lungenabszess), andere Infektionen, MRE und alternative Diagnosen aktiv auszuschließen.
- Nach Erregeridentifikation und Stabilisierung der klinischen Situation (Therapieansprechen) sollte die Therapie frühzeitig deeskaliert oder fokussiert werden [15, 16].
- Eine Kombinationstherapie ist in der Regel dann nur noch im Einzelfall bei hochgradig resistenten Gram-negativen Erregern sinnvoll.
- Die Therapiedauer beträgt in der Regel acht Tage (Ausnahmen siehe unten).

**Table 1:** 

**Keine schwere Infektion, niedriges Risiko für MRE oder *Pseudomonas***		
**1. Wahl:** Ceftriaxon 1 × 2 g/d Cefotaxim 3 × 2 g/d	**2. Wahl:** Levofloxacin^a^ 2 × 500 mg/d oder Moxifloxacin^a^ 1 × 400 mg/d Ampicillin/Sulbactam^b^ 3 × 3 g/d Amoxicillin/Clavulansäure^b^ 3 × 2,2 g/d Ertapenem 1 × 1 g/d	
**Keine schwere Infektion, niedriges Risiko für MRE aber hohes Risiko für *Pseudomonas, beziehungsweise*** **VAP, schwere HAP oder Sepsis (kein septischer Schock), niedriges Risiko für MRE aber auch niedriges Risiko für** ***Pseudomonas***		
**1. Wahl:** Piperacillin/Tazobactam 3 × 4,5 g/d (bei Mukoviszidose 4 × 4,5 g/d)	**2. Wahl:** Cefepim 3 × 2 g/d Meropenem 3 × 1 g/d (bei Mukoviszidose 3 × 2 g/d)	
**VAP, schwere HAP oder Sepsis UND hohes Risiko für MRE oder *Pseudomonas***		
**1. Wahl:** Piperacillin/Tazobactam 3–4 × 4,5 g/d Ceftazidim^c^ 3 × 2 g/d Cefepim 3 × 2 g/d Meropenem 3 × 2 g/d (bevorzugt bei lokaler ESBL^d^-Inzidenz > 10 %)	**In Kombination mit:** Ciprofloxacin^c^ 3 × 400 mg/d oder Levofloxacin 2 × 500 mg/d oder Aminoglykosid	**Bei MRSA-Verdacht oder lokaler MRSA-Inzidenz von >** **10** **% zusätzlich:** Linezolid 2 × 600 mg/d oder Vancomycin 2 × 15 mg/kg KG/d (Talspiegel 15–20 μg/ml oder AUC/MHK 400–600 mg/h/L)
**VAP, schwere HAP oder Sepsis UND lokale Inzidenz für MDR-*Pseudomonas* oder *Klebsiella pneumoniae* Carbapenemase (KPC**^**5**^**)** **>** **5** **%**		
**1. Wahl:** Ceftolozan/Tazobactam^c^ 3 × 2 g/1 g *(bei hoher MDR-Pseudomonas-Inzidenz)* Ceftazidim-Avibactam^c^ 3 × 2 g/0,5 g *(bei hoher KPC*^*e*^*-Inzidenz)*	**In Kombination mit:** Ciprofloxacin^c^ 3 × 400 mg/d oder Levofloxacin 2 × 500 mg/d oder Aminoglykosid	**Bei V.** **a. MRSA oder lokaler MRSA-Inzidenz von >** **10** **% zusätzlich:** Linezolid 2 × 600 mg/d oder Vancomycin 2 × 15 mg/kg KG/d (Talspiegel 15–20 μg/ml oder AUC/MHK 400–600 mg/h/L)
**Empirische Initialtherapie bei Immunsuppression und Risiko für opportunistische Infektionen**		
– Antibakterielle Therapie wie oben – Zusätzlich unverzügliche fungale, virologische und mykobakterielle Diagnostik (siehe Teil 1) – Bei schwerem Krankheitsbild und hochgradigem Verdacht auf *Pneumocystis jirovecii*-Pneumonie, invasive Schimmelpilz- oder CMV-Infektion empirische Therapie bis zum Ausschluss der entsprechenden Infektion gerechtfertigt		
**RISIKOFAKTOREN für das Vorhandensein von *Pseudomonas aeruginosa*** (das Risiko für eine *Pseudomonas*-HAP steigt mit der Zahl der erfüllten Faktoren und der Schwere der Erkrankung)		
– Bronchiektasen – Fortgeschrittene chronisch obstruktive Lungenerkrankungen – Vorbekannte chronische *Pseudomonas*-Infektion/-Kolonisation		
**RISIKOFAKTOREN für das Vorhandensein von MRE** (das Risiko für eine MRE-Infektion steigt mit der Zahl der erfüllten Faktoren und der Schwere der Erkrankung)		
– Lokale MRE-Rate von > 10 % – Antimikrobielle Breitspektrum-Therapie in den letzten 3 Monaten – Hospitalisierung ≥ 5 Tage (late-onset) – Vorbekannte MRE-Kolonisation/-Infektion in den letzten 12 Monaten – Medizinische Versorgung in Süd- und Osteuropa, Afrika, Naher Osten, Asien in den letzten 3 Monaten – Septischer Schock, ARDS, Sepsis-assoziierte Organdysfunktion, Behandlung auf ICU ***Zusätzliche Risikofaktoren bzgl. MRSA*** *(kein validierter MRSA-Risiko-Score für HAP oder Sepsis verfügbar):* – Vorbekannte MRSA-Kolonisation oder Z. n. MRSA-Infektion, ICU-Aufenthaltsdauer > 6 d, invasive maschinelle Beatmung, rezente Therapie mit Fluorchinolonen oder Cephalosporinen, zentrale Venenverweilkatheter (beispielsweise Dialyse), rezent stattgehabte Operation (beispielsweise Neurochirurgie)		
**RISIKOFAKTOREN für das Vorhandensein opportunistischer HAP-Erreger **(in erster Linie *Pneumocystis jirovecii*, Schimmelpilze, CMV, HSV‑1, Mykobakterien)		
– Organ- oder Knochenmark- bzw. Stammzelltransplantation – Chemotherapie solider oder hämatologischer Neoplasien mit und ohne Neutropenie – HIV im Stadium AIDS – Immunsuppressive oder immunmodulierende Therapie bei Autoimmunopathien – Systemische Glukokortikoidtherapie über einen Zeitraum von mindestens 4 Wochen mit einer Erhaltungsdosis ≥ 10 mg/d Prednisolon-Äquivalent (bei ICU-Patienten mit schwerer Influenza- bzw. SARS-CoV‑2 Pneumonie oder mit sehr hoher Glukokortikoiddosis auch bereits ab 1 Woche)		

*AIDS* „acquired immune deficiency syndrome“, *ARDS* „acute respiratory distress syndrome“, *AUC* „area under the curve“, *CMV* Cytomegalovirus, *ESBL* „extended spectrum β‑lactamase“, *HAP* „hospital-acquired pneumonia“, *HIV* „human immunodeficiency virus“, *HSV* Herpes simplex Virus, *ICU* „intensive care unit“, *KG* Körpergewicht, *KPC* Klebsiella pneumoniae carbapenemase, *MDR* „multidrug resistant“, *MHK* minimale Hemmkonzentration, *MRE* multiresistente Erreger, *MRSA* Methicillin-resistente Staphylococcus aureus, *SARS-CoV* „severe acute respiratory syndrome coronavirus“, *VAP* „ventilator-associated pneumonia“

^a^Rote Hand Brief bezüglich möglicher Nebenwirkungen

^b^Im Gram-negativen Bereich in Österreich oft schon Resistenzraten von > 10 %

^c^Nicht ausreichend wirksam im Gram-positiven Bereich (z. B. bei *Staphylococcus aureus*). Kombination mit einer gegen Gram-positive Erreger wirksamen Substanz zu erwägen

^d^ESBL: in der Regel Resistenz gegenüber Cephalosporinen (inklusive Cefepim und Ceftazidim)

^e^KPC: in der Regel Resistenz gegenüber Cephalosporinen und Carbapenemen

### Wichtig.

Folgende Regeln sind zusätzlich bei VAP, schwerer HAP, ICU-HAP oder Sepsis zu beachten:

- Werden die Kriterien einer Sepsis erfüllt (= medizinischer Notfall) oder wird eine ICU-HAP oder VAP diagnostiziert, gilt als Faustregel, die antiinfektive Therapie idealerweise innerhalb der ersten Stunde einzuleiten [17, 18].
- Die Therapie erfasst initial ein breites Erregerspektrum (hit hard, broad and early). Liegt zusätzlich ein hohes MRE- oder *Pseudomonas*-Risiko vor, sollte empirisch mit einer Kombinationstherapie begonnen werden.
- Die Dosierung der Antiinfektiva sollte nach pharmakokinetischen/-dynamischen Prinzipien optimiert werden (beispielsweise Betalaktam-Antibiotika nach initialer Bolus-Gabe als prolongierte Infusion verabreichen, siehe auch unten). Bei bestimmten Substanzen ist ein Drug-Monitoring sinnvoll.
- Bei schwerer Immunsuppression (Tab. 1), aber auch bei ICU-pflichtiger Influenza- oder SARS-CoV-2-Pneumonie („severe acute respiratory syndrome coronavirus“), wird primär auch Pilzdiagnostik veranlasst (in der Regel CT-Thorax, Kulturen, Serum- und BAL-Galactomannan, Serum-(1-3)-beta-D-Glucan, gegebenenfalls auch PCR; BAL: bronchioalveoläre Lavage). Bei hohem Risiko für eine invasive Pilzinfektion (Candidämie, *Pneumocystis*-Pneumonie, invasive Aspergillose) und schwerem Krankheitsbild sollte bis zum Ausschluss empirisch auch eine antifungale Therapie eingeleitet werden.

Österreich und Deutschland gelten noch nicht als klassische Hochinzidenzländer für MRE. Wie bereits im Teil 1 erwähnt, stagnieren aktuell die nosokomialen MRE-Raten und sind teils sogar leicht rückläufig. Somit können in Österreich (bei sonst fehlenden individuellen MRE-Risikofaktoren und günstiger lokaler epidemiologischer Situation) zur empirischen HAP-Therapie auf Normalstation in der Regel traditionelle antimikrobielle Substanzen verwendet werden (orientierend siehe Tab. 1).

### Wichtig.

Da in Österreich lokal in einzelnen Krankenhäusern oder in Risikobereichen (beispielsweise Stationen mit hohem Anteil an Bronchiektasen-Patient*innen oder Lungentransplantierten, hämatologische Stationen, ICU) höhere MRE-Raten auftreten können, ist eine regelmäßige Information der klinisch tätigen Ärztinnen und Ärzte durch das zuständige mikrobiologische Labor – beispielsweise in Form von jährlichen Resistenzberichten – zu empfehlen.

Nebenwirkungsreiche MRE-Reservesubstanzen (wie Colistin, Aminoglykoside, Linezolid, Glykopeptide), kostenintensive oder die in den letzten Jahren zugelassenen „neuen“ Antibiotika (Tab. 2) sollten nur bei relevantem MRE-Risiko, hoher lokaler Resistenzrate, Nachweis eines MRE oder wenn traditionelle Substanzen kontraindiziert bzw. in vitro unwirksam sind, zum Einsatz kommen (Tab. 1 und 3). Dieses Szenario ist in Österreich besonders in den oben genannten Risikobereichen relevant, da hier ein höherer Anteil an MRE-Infektionen im Vergleich zu Normalstationen zu finden ist. Die Förderung von Antibiotika-Stewardship-Programmen kann in diesem Setting den Antibiotikaverbrauch und damit assoziierte Kosten signifikant reduzieren [19].

**Table 2:** 

Substanz	Spektrum im MDR-Bereich	Tagesdosis	Kommentar
**Ceftobiprol** (Zevtera®)	MRSA	3 × 500 mg über je 2 h	Für HAP, aber nicht für VAP zugelassen Auch geeignet für non-MDR *P. aeruginosa*
**Ceftolozan-Tazobactam** (Zerbaxa®)	ESBL AmpC CR* P. aeruginosa *(non-MBL)	3 × 2 g/1 g über je 1 h	Nicht ausreichend wirksam im Gram-positiven Bereich (z. B. bei MSSA)
**Meropenem-Vaborbactam** (Vabomere®)	ESBL AmpC KPC	3 × 2 g/2 g über je 3 h	Auch geeignet für non-MDR *P. aeruginosa* Auch geeignet für non-MDR *A. baumannii*
**Imipenem-Relebactam Cilastatin** (Recarbrio®)	ESBL AmpC KPC CR *P. aeruginosa *(non-MBL)	4 × 0,5 g/0,25 g/0,5 g über je 30 min	*–*
**Ceftazidim-Avibactam** (Zavicefta®)	ESBL AmpC KPC OXA 48 CR *P. aeruginosa *(non-MBL)	3 × 2 g/0,5 g über je 2 h	Nicht ausreichend wirksam im Gram-positiven Bereich (z. B. bei MSSA)
**Cefiderocol** (Fetcroja®)	ESBL AmpC KPC MBL CR *P. aeruginosa* (CR *A. baumannii)* *S. maltophilia*	3 × 2 g über je 8 h	„All-cause“ Letalität für Patient*innen mit CR- *A. baumannii*-Infektionen und Cefiderocol-Monotherapie möglicherweise erhöht Nicht ausreichend wirksam im gram-positiven Bereich (z. B. bei MSSA)

*AmpC* AmpC β‑lactamases (Betalaktamasen der Klasse C, Resistenz gegenüber Cephalosporinen), *CR* „carbapenem-resistant“, *CRE* „carbapenem-resistant enterobacterales“, *ESBL* „extended spectrum β‑lactamase“ (Betalaktamasen der Klasse A/D, Resistenz gegenüber Cephalosporinen inklusive Cefepim und Ceftazidim), *HAP* „hospital acquired pneumonia“, *KPC* Klebsiella pneumoniae carbapenemase (Betalaktamasen der Klasse A, Resistenz gegenüber Cephalosporinen und Carbapenemen), *MBL* „metallo-beta-lactamases“ (Betalaktamasen der Klasse B, Resistenz gegenüber Carbapenemen, Aztreonam ist wirksam, Subgruppen: NDM, VIM, IMP), *MDR* „multidrug-resistant“, *MRSA* Methicillin-resistente Staphylococcus aureus, *MSSA* Methicillin-sensible Staphylococcus aureus, *OXA* OXA „β‑lactamases“ (Betalaktamasen der Klasse D, Resistenz gegenüber Carbapeneme), *VAP* „ventilator-associated pneumonia“

**Table 3:** 

Erreger und Resistenz	Empfehlung entsprechend der vorliegenden Resistenz	Grad der Empfehlung
**Enterobakterien**		
**ESBL**	**Leichte Infektion:** Traditionelle (alte) Substanzen (z. B. Piperacillin/Tazobactam, Amoxicillin/Clavulansäure, Fluorchinolone bei In-vitro-Wirksamkeit)	Schwach
	**Sepsis oder Hochrisiko-Infektionen**^**a**^**:** Meropenem oder Imipenem (bei klinischer Stabilisierung im Verlauf Deeskalation nach Antibiogramm)	Stark
**Carbapenem-Resistenz (CRE)**	**Leichte Infektion:** Traditionelle Substanzen (bei In-vitro-Wirksamkeit)	Schwach
	**Sepsis: **Meropenem/Vaborbactam oder Ceftazidim/Avibactam	Schwach
	Tigecyclin^b^ nach Möglichkeit nicht (oder nur high-dose) einsetzen	Schwach
**Breite Carbapenem-Resistenz (X-CRE)**	Cefiderocol (alternativ Aztreonam plus Ceftazidim/Avibactam)	Schwach
**Kombinationstherapie bei CRE-Infektionen** – bei guter Empfindlichkeit in der Regel nicht notwendig – bei Sepsis empfohlen, wenn Erreger nur noch auf Colistin, Aminoglykoside, Tigecyclin^b^ oder Fosfomycin sensibel ist bzw. neue BLBLI nicht verfügbar sind (Einsatz von mehr als einer aktiven Substanz empfohlen) – bei einer Meropenem-MHK ≤ 8 mg/L kann eine Carbapenem Kombinationstherapie mit high-dose extended-Carbapenem-Infusion erwogen werden		
***Pseudomonas aeruginosa***		
**Keine Carbapenem-Resistenz**	Bei In-vitro-Wirksamkeit sollten traditionelle (alte) *Pseudomonas*-wirksame non-Carbapenem-β-Laktame (z. B. Piperacillin/Tazobactam, Ceftazidim, Cefepim, Aztreonam) gegenüber Carbapenemen bevorzugt werden	GPS
**Carbapenem-Resistenz**	**Leichte Infektion:** Traditionelle *Pseudomonas*-wirksame Substanzen (bei In-vitro-Wirksamkeit)	GPS
	**Sepsis oder Hochrisiko-Infektionen**^**a**^**:** Ceftolozan/Tazobactam (alternativ bei In-vitro-Wirksamkeit traditionelle *Pseudomonas*-wirksame non-Carbapenem-β-Laktame als high-dose-extended infusionstherapie oder Imipenem/Relebactam oder Ceftazidim/Avibactam)	Schwach
**Kombinationstherapie bei *Pseudomonas*-Infektionen** – bei guter Empfindlichkeit in der Regel nicht notwendig – bei Sepsis empfohlen, wenn Erreger nur auf Colistin, Aminoglykoside oder Fosfomycin sensibel ist bzw. neue BLBLI nicht verfügbar sind – bezüglich der neuen BLBLI oder Cefiderocol kann aktuell keine Empfehlung für oder gegen eine Kombinationstherapie ausgesprochen werden (fehlende Evidenz)		
***Acinetobacter baumannii***		
**Keine Carbapenem-Resistenz**	**Leichte Infektion:** Ampicillin/Sulbactam, Meropenem, Cefepim als Monotherapie **Schwere Infektion: **Kombinationstherapie	GPS
**Carbapenem-resistenter ***A. baumannii* **(CRAB)**	**Sulbactam empfindliche CRAB**: Ampicillin/Sulbactam	Schwach
	**Sulbactam resistente CRAB: **Colistin oder high-dose Tigecyclin^b^ (bei In-vitro-Wirksamkeit)	Schwach
	CRAB sollten nicht primär mit Cefiderocol behandelt werden (nur bei fehlendem Ansprechen auf andere Substanzen oder Unverträglichkeit)	Schwach
**Kombinationstherapie bei schweren CRAB-Infektionen** – Bei Sepsis wird eine Kombination von zwei in vitro wirksamen Substanzen empfohlen (high-dose Ampicillin/Sulbactam, Carbapenem, Colistin, Aminoglykosid, Minocyclin, high-dose Tigecyclin^b^, Rifampicin). – Nicht empfohlen ist jedoch die Kombination von Colistin-Meropenem oder Colistin-Rifampicin (in Studien kein Vorteil gegenüber der Monotherapie) – Bei einer Meropenem-MHK ≤ 8 mg/L kann eine Carbapenem Kombinationstherapie mit High-dose extended-Carbapenem-Infusion erwogen werden		
***Stenotrophomonas maltophilia***		
**Leichte Infektion:** Primär Trimethoprim/Sulfamethoxazol, Minocyclin oder Levofloxacin als Monotherapie (bei fehlender klinischer Besserung als Kombinationstherapie)		
**Schwere Infektion:** Trimethoprim/Sulfamethoxazol PLUS Minocyclin, (alternativ zu Minocyclin: Levofloxacin, Tigecyclin^b^, Cefiderocol), alternativ Ceftazidim/Avibactam PLUS Aztreonam		

*BLBLI* „β-lactam/β-lactamase inhibitor“, *CR* „carbapenem-resistant“, *CRE* „carbapenem-resistant enterobacterales“, *ESBL* extended spectrum β‑lactamase (Betalaktamasen der Klasse A/D, Resistenz gegenüber Cephalosporinen inklusive Cefepim und Ceftazidim), *GPS* „good practice statement“, *MHK* minimale Hemmkonzentration, *X‑CRE* CRE mit MBL oder breite Resistenz gegen alle anderen einsatzfähigen Substanzen (inkl. Ceftazidim/Avibactam oder Meropenem/Vaborbactam)

^a^Hochrisiko-Infektion klassifiziert nach dem INCREMENT Score

^b^CAVE: Tigecyclin nicht für Pneumonie zugelassen

## Prolongierte Infusionsdauer bei schwerer HAP

Bei schwerer HAP und/oder Infektion mit Gram-negativen Erregern mit grenzwertig erhöhter MHK (minimale Hemmkonzentration) ist für Breitband-Betalaktam-Antibiotika wie beispielsweise Piperacillin/Tazobactam, Meropenem, Ceftazidim oder Cefepim nach initialer Bolus-Gabe eine Verlängerung der Infusionsdauer auf mehrere Stunden zur pharmakokinetischen Verbesserung der Wirksamkeit empfehlenswert. Zusätzlich sollte bei kritisch kranken Patient*innen ein Drugmonitoring erfolgen [24]. Hierdurch kann ein im Vergleich zur üblichen Kurzinfusion deutlich besseres Ansprechen beispielsweise von *Pseudomonas*-wirksamen Betalaktam-Antibiotika mit einer Reduktion der Mortalität von 30 % erreicht werden [25].

## Allgemeine Therapiedauer

Bei gutem Therapieansprechen (klinischer und laborchemischer Besserung), unkompliziertem Verlauf und empfindlichem Erreger sollte die Therapiedauer auch bei *MRSA- und Acinetobacter-spp.-*assoziierter HAP/VAP leitliniengerecht 7–8 Tage nicht überschreiten [1, 2].

### Wichtig.

Unter folgenden Umständen kann eine Verlängerung der Therapiedauer bei bakterieller HAP dennoch empfehlenswert sein [6, 26]:

- schwere Bronchiektasenerkrankung
- schwere Immunsuppression
- Empyem, Lungenabszess, einschmelzende Pneumonien oder superinfizierte Kavernen
- verzögertes Therapieansprechen (nur langsam regrediente Entzündungsparameter)
- initial inadäquate antimikrobielle Therapie
- Nachweis einer Bakteriämie durch Gram-negative Erreger oder *Staphylococcus-aureus*
- Nachweis von „extensively drug resistant“ (XDR)/„pandrug resistant“ (PDR) oder Carbapenem-resistenten Enterobakterien

Ob bei einer VAP mit Nachweis von *Pseudomonas* die Therapiedauer generell von 8 auf 15 Tage prolongiert werden soll, ist unverändert umstritten. Eine aktuelle randomisiert, kontrollierte, multizentrische Studie mit 186 ICU-Patient*innen bestätigte jedoch erneut, dass eine lediglich 8‑tägige Antibiose die Rezidivrate bei einer *Pseudomonas*-VAP erhöht und auch mit einem tendenziellen Anstieg der Mortalität assoziiert ist [27, 28].

### Wichtig.

Letztendlich kann bei HAP/VAP die Entscheidung zur Beendigung einer antiinfektiven Therapie auch unter Berücksichtigung des Procalcitonin-Verlaufs getroffen werden. Mehrere Studien haben gezeigt, dass bei gutem Therapieansprechen und einem Procalcitonin < 0,25 ng/ml oder einer Procalcitonin-Reduktion von ≥ 80 % die antiinfektive Therapie beendet werden kann [29].

## Therapie spezieller Erreger-Gruppen

Im Folgenden wird die Therapie schwer zu behandelnder bakterieller HAP-Erreger (MRE oder potenzielle MRE) in Grundzügen umrissen. Details können in den jeweils angegebenen Literaturquellen nachgelesen werden. Aus Platzgründen wird in diesem Artikel nicht weiter auf die Therapie wichtiger opportunistischer HAP-Erreger wie *Pneumocystis jirovecii, Aspergillus spp., *Zytomegalievirus (CMV) und Herpes-Simplex-Virus(HSV)-1 eingegangen.

**Methicillin-resistente *S. aureus*: **Bei einer lokalen MRSA-Rate von > 10–20 % (in Österreich auf Normalstationen in der Regel nicht der Fall) sollte das Spektrum der empirischen HAP-Therapie unter Berücksichtigung der Schwere der Infektion auch MRSA erfassen. Zusätzlich sind einerseits spezifische MRSA-Risikofaktoren (Tab. 1) und andererseits Risiken einer ungerechtfertigten MRSA-Therapie (erhöhte Mortalität, Nebenwirkungen, Resistenzentwicklung) abzuwägen [14]. Empirisch wird die konventionelle HAP/VAP-Therapie um Linezolid oder Vancomycin erweitert [30]. Ceftobiprol ist nur für CAP und HAP, jedoch nicht für VAP zugelassen.

### Wichtig.

Die genannten „first-line“-Medikamente haben relevante Nebenwirkungsprofile, weshalb eine MRSA-Infektion unverzüglich ausgeschlossen (Kultur und wenn möglich auch PCR aus Nasenabstrich, Sputum/BAL) und das MRSA-Antibiotikum so frühzeitig wie möglich wieder abgesetzt werden sollte [31].

Die Wirksamkeit von Linezolid, Vancomycin und Ceftobiprol zur Behandlung einer MRSA-HAP kann als gleichwertig angesehen werden [32, 33]. Bei der VAP ist Ceftobiprol der Kombination von Ceftazidim/Linezolid jedoch unterlegen [34]. Im Einzelfall wird jenes Medikament bevorzugt, welches mit Hinblick auf Begleiterkrankungen und die aktuelle klinische Situation das vermutlich geringste Nebenwirkungsrisiko mit sich bringt. Bei Linezolid sind in erster Linie gastrointestinale Nebenwirkungen, bei längerer Therapiedauer Thrombopenien und Neuropathien häufig. Vancomycin verursacht vordergründig renale Funktionsstörungen, welche dosisabhängig und besonders häufig in Kombination mit Piperacillin-Tazobactam beobachtet werden [35, 36]. Bei Therapie mit Vancomycin sind daher die regelmäßige Kontrolle der Nierenfunktion und ein intensiviertes Drugmonitoring obligat. Bezüglich Letzterem wurden bisher in erster Linie Talspiegelbestimmungen empfohlen. Inzwischen wird jedoch zunehmend die komplexere Bestimmung der AUC/MIC-Ratio („area under the curve“/„minimal inhibitory concentration“) gefordert (siehe Empfehlung der Infectious Diseases Society of America von 2020 [37]). Teicoplanin wird gelegentlich als weniger nephrotoxische Alternative zu Vancomycin diskutiert. Es liegen für Teicoplanin jedoch nur wenige MRSA-HAP-Studienergebnisse vor und Teicoplanin penetriert Lungengewebe schlechter als Vancomycin.

Als MRSA-Antibiotika der zweiten Wahl können bei einer HAP Tedizolid und Telavancin erwogen werden. Andere MRSA-Antibiotika kommen in der Regel nicht in Betracht (für Clindamycin, Doxycyclin, Trimethoprim/Sulfamethoxazol liegen nur wenige klinische Daten zur MRSA-HAP vor; Daptomycin wird von Surfactant inaktiviert; Ceftarolin und Tigecyclin sind für die HAP nicht zugelassen).

**ESBL-produzierende Enterobakterien, inkl. Pseudomonaden (resistent gegen Cephalosporine wie Ceftriaxon, Ceftazidim, Cefepim, Ceftobiprol): **Bei einer lokalen ESBL-Rate von > 10 % sollte eine schwere HAP/VAP in erster Linie mit einem Carbapenem (Meropenem, Imipenem, Ertapenem) therapiert werden. Auch Ceftazidim/Avibactam, Ceftolozan/Tazobactam und Cefiderocol kommen bei ESBL-HAP/VAP in Betracht, sollten jedoch primär bei Carbapenem-Resistenz und speziell bei Carbapenemase-produzierenden Erregern zum Einsatz kommen (siehe unten und Tab. 1, 2 und 3).

**Carbapenemase-produzierende MRE (CP-MRE), inkl. CP-Pseudomonaden: **Eine Carbapenem-Resistenz (CR) kann nicht nur durch Carbapenemasen (Serin-Carbapenemase = Ampler-Klasse A [u. a. KPC] und D [OXA-Subtypen], Metallo-Carbapenemase = Ampler-Klasse B [NDM, VIM, IMP]) sondern auch durch non-enzymatische Resistenzmechanismen (beispielsweise durch Mutationen in Porin-kodierenden Genen oder gesteigerte Expression von Efflux-Pumpen) bedingt sein [38].

### Wichtig.

Um bei einer In-vitro-CR das Vorliegen einer Carbapenemase zu bestätigen (und auch einen Überblick bezüglich der lokalen CP-MRE-Rate zu erhalten), sind weiterführende phenotypische, genotypische oder immunchromatografische Untersuchungen notwendig und empfohlen [39].

Die Evidenz zur Behandlung einer CP-MRE-HAP ist begrenzt. Liegt eine CP-MRE-HAP vor, sollte die Therapie unter Berücksichtigung der In-vitro-Sensibilitätstestung zusammen mit MRE-Expert*innen festgelegt werden. Bei nachgewiesener In-vitro-Wirksamkeit können primär weiterhin auch traditionelle Substanzen wie Ceftazidim oder Cefepim verwendet werden. Ist dies nicht der Fall, kommen die neuen MDR-Reserveantibiotika Ceftolozan/Tazobactam, Ceftazidim/Avibactam, Meropenem/Vaborbactam, Imipenem/Relebactam/Cilastatin oder Cefiderocol zum Einsatz (Tab. 1 und 2 und 3). Cefiderocol, ein Siderophor-Cephalosporin, gilt aufgrund seines extrem breiten Wirkspektrums als besonders exklusives Reserveantibiotikum und sollte in erster Linie nur bei MDR-Erregern mit zusätzlich auch sehr breiter Carbapenem-Resistenz zum Einsatz kommen. Colistin ist in vielen Fällen ebenfalls wirksam, aber das Nebenwirkungsprofil ist im Vergleich zu den genannten Alternativen ungünstig.

***Acinetobacter baumannii***: Bei Nachweis von *A. baumannii* ist klinisch zunächst zwischen einer Kolonisation und einer behandlungspflichtigen Infektion zu unterscheiden. Wird von einer relevanten Infektion ausgegangen, richtet sich die Therapie ebenfalls nach der lokalen Resistenzsituation und der In-vitro-Austestung. Bei nachgewiesener Empfindlichkeit kommt primär Ampicillin/Sulbactam zum Einsatz, alternativ ein Carbapenem [40]. Bei einer schweren Infektion oder multiresistentem *A. baumannii* sollte eine Kombinationstherapie angestrebt werden (Tab. 3). Bei In-vitro-Unempfindlichkeit für Ampicillin/Sulbactam kann bei Carbapenem-Resistenz high-dose Ampicillin/Sulbactam mit beispielsweise Colistin, Tigecyclin oder Minocyclin kombiniert werden. Auch inhalatives Colistin in Kombination mit anderen Antibiotika kommt in Betracht [41].

Cefiderocol ist bei den meisten MDR-*A.-baumannii*-Stämmen in vitro wirksam. In einer randomisierten Phase-III-Studie war Cefiderocol bei schweren Infektionen mit CR-MRE im Vergleich zur bestmöglichen Therapie mit anderen Antibiotika formell ähnlich gut wirksam, aber numerisch traten in der Cefiderocol-Gruppe mehr Todesfälle auf, besonders bei Patient*innen mit einer *A.-baumannii-*Infektion. Somit sollte Cefiderocol bei *A.-baumannii-*Infektionen vorerst nur verwendet werden, wenn keine anderen Optionen zur Verfügung stehen [42].

***Stenotrophomonas maltophilia: ***Wie bei *A. baumannii* ist auch bei Nachweis von *S. maltophilia* klinisch zwischen einer Kolonisation und einer behandlungspflichtigen Infektion zu unterscheiden. Prinzipiell ist *S. maltophilia* per se ein MRE. Mittel der Wahl ist Trimethoprim/Sulfamethoxazol (TMP/SMX). Bei Unverträglichkeit oder TMP/SMX-Resistenz ist Levofloxacin Mittel der 2. Wahl (weniger Daten liegen für Moxifloxacin vor). Bei schweren Infektionen wird eine Kombinationstherapie empfohlen (Tab. 3).

## Inhalative Antibiotika

Der Einsatz inhalativer Antibiotika (iAB) bei der HAP und speziell bei der VAP wird seit vielen Jahren intensiv diskutiert und wurde in einer Vielzahl von Studien untersucht. Im Jahr 2017 sprach sich die European Society of Clinical Microbiology and Infectious Diseases (ESCMID) gegen die routinemäßige Verwendung von iAB bei invasiv-beatmeten Patient*innen aus (Evidenz der klinischen Effektivität im Vergleich zur Standardtherapie gering und Nebenwirkungsrisiko, in erster Linie respiratorische Komplikationen durch Bronchospasmus, in den vorliegenden Studien womöglich unterschätzt) [43]. Die französische HAP-Leitlinie empfiehlt 2018 für ICU-Patient*innen die Anwendung von inhalativem Colistin oder Aminoglykosiden als Option für in vitro empfindliche MRE-Infektionen (in erster Linie als Monotherapie: „substitution rather than adjunctive therapy“), wenn keine anderen Antibiotika alternativ verwendet werden können [44].

Seither wurden zwei weitere randomisiert-kontrollierte Phase-II/III-Studien veröffentlicht. Sie zeigten keine Reduktion der Mortalität im Vergleich zur intravenösen Standardtherapie, wobei beide Studien methodische Schwächen aufwiesen (beispielsweise nur 50 % der Patient*innen hatten eine MRE-Infektion) [45]. Aktuell sind methodisch besser konzipierte Studien in Arbeit, welche den Einsatz iAB ausschließlich bei nicht-bakteriämischer VAP mit Gram-negativen MRE und unter optimalen Ventilator-Einstellungen untersuchen werden [46, 47].

Bis zum Vorliegen der Studienergebnisse sollten iAB in der klinischen Routine nur in begründeten Ausnahmefällen bei VAP mit komplizierten MRE-Infektionen zum Einsatz kommen.

## DFP-Fragen

Antibiotika der 1. Wahl für die Therapie einer HAP auf Normalstation *ohne* Risikofaktoren für multiresistente Erreger oder *Pseudomonas aeruginosa* sind … (eine Antwort richtig)

Piperacillin/Tazobactam.

Amoxicillin/Clavulansäure.

Ceftriaxon.

Azithromycin.

Nennen Sie zwei Grundregeln für die empirischen HAP-Therapie. (zwei Antworten richtig)

Bei HAP sollte initial immer eine antiinfektive Kombinationstherapie eingeleitet werden, die MRSA und ESBL erfasst. Erst nach mikrobiologischem Ausschluss multiresistenter Erreger kann die Therapie als Monotherapie fortgesetzt werden.

Die Therapiedauer beträgt in der Regel 8 Tage.

Die mikrobiologische Diagnostik sollte spätestens 12 h nach Beginn oder Umstellung der antimikrobiellen Therapie abgenommen werden.

Bei septischem Zustandsbild ist die Einleitung einer antimikrobiellen Therapie innerhalb der 1. Stunde wünschenswert („hit hard and early“).

Eine Verlängerung der Infusionsdauer bei schwerer HAP … (eine Antwort richtig)

kann die pharmakokinetische Wirksamkeit verdoppeln.

verbessert das Ansprechen auf gram-negative Erreger wie *P. aeruginosa* im Vergleich zur Kurzinfusion.

ermöglicht eine Dosisreduktion von 30 % bei Niereninsuffizienz.

sollte nach neuen HAP-Guidelines unbedingt vermieden werden.

Nennen Sie Medikamente der 1. Wahl für die Therapie einer MRSA-HAP bei nicht-beatmeten Patient*innen. (drei Antworten richtig)

Vancomycin

Linezolid

Daptomycin

Ceftobiprol

Welche Aussagen zu *Acinetobacter baumannii* sind richtig? (zwei Antworten richtig)

Eine Infektion mit *Acinetobacter baumannii* muss in jedem Fall antibiotisch behandelt werden.

Die Therapie der 1. Wahl ist Ampicillin/Sulbactam.

Es muss zwischen einer Kolonisation und einer behandlungspflichtigen Infektion unterschieden werden.

Die Therapie der 1. Wahl ist Trimethoprim/Sulfamethoxazol.

Antibiotika der 1. Wahl für die Therapie einer VAP oder schweren HAP bei zusätzlich hohem Risiko für MRE oder *Pseudomonas* sind … (vier Antworten richtig)

Meropenem.

Tigecyclin.

Ceftolozan/Tazobactam (bei hoher MDR-*Pseudomonas*-Inzidenz).

Ceftazidim-Avibactam (bei hoher KPC-Inzidenz).
